# Detection of *Trichomonas vaginalis* Using the Polymerase Chain Reaction in Pregnant and Non-Pregnant Women

**DOI:** 10.1155/S1064744994000335

**Published:** 1994

**Authors:** J. Jeremias, D. Draper, M. Ziegert, W. Jones, S. Inglis, J. A. McGregor, S. S. Witkin

**Affiliations:** 1 Department of Obstetrics and Gynecology Cornell University Medical College 515 East 71st Street New York NY 10021 USA; 2 Department of Obstetrics and Gynecology University of Colorado Health Science Center Denver CO USA

## Abstract

*Objective:* *Trichomonas vaginalis* vaginal infections are often both asymptomatic and difficult to detect by current methods. We evaluated the ability of a newly developed polymerase chain reaction (PCR) assay to identify *T. vaginalis* in vaginal samples from pregnant and non-pregnant women.

*Methods:* In the 1st study, we compared the prevalence of *T. vaginalis* detection by PCR and culture using Diamond's medium in 52 women with symptoms of vaginal infection. In the 2nd study, *T. vaginalis* was detected using PCR and wet mount microscopy in 131 asymptomatic pregnant women.

*Results:* Among the women with symptoms of vaginitis, 7 (13.5%) were PCR-positive for *T. vaginalis*. Six of the PCR-positive women, but none of the PCR-negative women, were culture-positive for this organism. All but 1 of the women with candidal vaginitis or bacterial vaginosis were PCR-negative for T. *vaginalis*. Among the asymptomatic pregnant women, all of whom were negative for *T. vaginalis* by wet mount, l0 (7.6%) were PCR-positive for *T. vaginalis*.

*Conclusions:* PCR offers a rapid and sensitive alternative to culture and microscopy for the detection of *T. vaginalis* vaginal infections in both symptomatic and asymptomatic women.


Infectious Diseases in Obstetrics and Gynecology 2:16–19 (1994)
© 1994 Wiley-Liss, Inc.

### Detection of *Trichomonas vaginalis* Using the Polymerase Chain Reaction in Pregnant and Non-Pregnant Women

**J. Jeremias, D. Draper, M. Ziegert, W. Jones, S. Inglis, J.A. McGregor, and S.S. Witkin**

*Department of Obstetrics and Gynecology, Cornell University Medical College, New York, NY (J.J., M.Z., S.I., S.S.W.), Department of Obstetrics and Gynecology, University of Colorado Health Science Center, Denver, CO (D.D., W.J., J.A.M.)*

### Abstract

***Objective:*** ***Trichomonas vaginalis*** **vaginal infections are often both asymptomatic and difficult to detect by current methods. We evaluated the ability of a newly developed polymerase chain reaction (PCR) assay to identify *T. vaginalis* in vaginal samples from pregnant and non-pregnant women.**

***Methods:*** **In the 1st study, we compared the prevalence of *T. vaginalis* detection by PCR and culture using Diamond's medium in 52 women with symptoms of vaginal infection. In the 2nd study, *T. vaginalis* was detected using PCR and wet mount microscopy in 131 asymptomatic pregnant women.**

***Results:*** **Among the women with symptoms of vaginitis, 7 (13.5%) were PCR-positive for *T. vaginalis*. Six of the PCR-positive women, but none of the PCR-negative women, were culture-positive for this organism. All but 1 of the women with candidal vaginitis or bacterial vaginosis were PCR-negative for T. *vaginalis*. Among the asymptomatic pregnant women, all of whom were negative for *T. vaginalis* by wet mount, 10 (7.6%) were PCR-positive for *T. vaginalis*.**

***Conclusions:*** **PCR offers a rapid and sensitive alternative to culture and microscopy for the detection of *T. vaginalis* vaginal infections in both symptomatic and asymptomatic women.**
© 1994 Wiley-Liss, Inc.

Key words
Vaginitis, pregnancy, trichomoniasis

*Trichomonas vaginalis*, a motile anaerobic protozoan, is a common sexually transmitted infectious pathogen worldwide.[1] Trichomoniasis is a frequent cause of vaginitis, exocervicitis, and urethritis. *T. vaginalis* infections are frequently asymptomatic and detectable only using culture techniques.[2,3] Recent studies implicate both symptomatic and asymptomatic *T. vaginalis* infections in the pathogenesis of preterm birth, preterm rupture of membranes,[4] and post-hysterectomy cuff infections.[5]

Despite the frequency of this infection, diagnosis of trichomoniasis remains problematic. The most common clinical method, direct microscopic examination of vaginal fluid ("wet preparation"), has a sensitivity of only 35–80%.[6] Vaginal or urethral culture using Diamond's medium has a sensitivity >90% for detecting *T. vaginalis*.[3,7] Some isolates, however, grow poorly or not at all in this medium.[7] In addition, the use of Diamond's medium is labor intensive, requiring daily examination for up to 7 days before the presence or absence of *T.*

**Address correspondence/reprint requests to Dr. Steven S. Witkin, Department of Obstetrics and Gynecology, Cornell University Medical College, 515 East 71st Street, New York, NY 10021.**

Received December 29, 1993

Accepted May 24, 1994

*vaginalis* can be definitively assessed.

Recently, Riley et al.[8] developed a polymerase chain reaction (PCR)-based assay for identification of *T. vaginalis*. Using primer pairs specific for a 102 base pair region of the *T. vaginalis* genome, they demonstrated that their assay readily detected a broad range of *T. vaginalis* isolates from different geographical regions of the United States. There was no cross-reactivity with human DNA or with DNA from a broad range of other eukaryotic or prokaryotic microorganisms. Testing their assay on a small number of vaginal samples, they detected *T. vaginalis* by PCR in 1 woman who was negative by wet mount.

In this study, we report on the further evaluation of this PCR assay to detect *T. vaginalis* in pregnant and non-pregnant women.

### SUBJECTS AND METHODS

### Subjects

The study groups consisted of 52 consecutive non-pregnant reproductive-age women with symptoms (discharge, burning, pruritis) of vaginitis evaluated at the University Hospital, Denver, CO, and 131 consecutive asymptomatic pregnant women seen at Cornell University Medical College, New York, NY. The pregnant women were between 15 and 40 years old with a singleton gestation <37 weeks and intact membranes.

### Microbial Analysis

At both sites, samples were obtained from the posterior fornix with sterile cotton swabs and utilized to prepare a slide for a microscopic "wet preparation" saline examination. In Colorado, a 2nd swab was obtained and used to inoculate modified Diamond's medium (Remel, Lenexa, KS). Cultures were incubated at 37°C in a 5% $CO_2$ atmosphere for 7 days. At daily intervals, beginning on day 2, aliquots were removed and examined microscopically for motile *T. vaginalis*. One swab was frozen at −70°C.

The presence of *Candida* in the vaginal samples was assessed by examining wet mounts in 10% KOH for the presence of pseudohyphae and/or budding yeast in 10 high power (×400) fields.

Bacterial vaginosis was diagnosed clinically when at least 3 of the following 4 criteria were met: pH > 4.5; thin homogeneous adherent discharge; presence of clue cells; amine odor on KOH wet mount.

### PCR Analysis

The procedure of Riley et al.[8] was utilized. Briefly, liquid was extracted from the previously frozen swab by squeezing with a sterile Pasteur pipette and transferred to a microcentrifuge tube. A pellet fraction was obtained and washed twice with phosphate-buffered saline (PBS). Lysis of *T. vaginalis* and exposure of its DNA were accomplished by resuspending the pellet in 0.25 ml Tris-HCl, pH 8.3, containing 50 mM KCl, 2.5 mM $MgCl_2$, 1% Brij 35 detergent, and 200 μg/ml proteinase K and incubating in a thermal cycler for 60 min at 56°C. The proteinase K was then inactivated by raising the temperature to 95°C for 10 min. Purified *T. vaginalis* from a symptomatic patient was always processed in parallel to the test samples. Buffer was processed as a negative control. Processed samples were stored at 4°C until tested.

For the analysis, 1-μl aliquots were mixed with 24 μl $H_2O$, heated at 94°C for 7 min to separate the DNA strands, and plunged into ice-$H_2O$ to prevent reannealing. An equal volume of reaction mixture was then added to yield 10 mM Tris-HCl, pH 8.3, 0.5 units *Taq* DNA polymerase, 1.5 mM $MgCl_2$, 50 mM KCl, 200 mM each dATP, dCTP, dGTP, and dTTP, and 2 oligonucleotide primer pairs specific for a 102 base pair region of the *T. vaginalis* genome. The samples were vortexed, microcentrifuged, and subjected to 40 cycles of sequential incubations at 94°C for 1 min, 47°C for 1 min, and 67°C for 1 min followed by a 7-min extension cycle at 67°C. A portion of the final reaction product was digested with *Hinfl* endonuclease as described.[7] The reaction products were subjected to electrophoresis on 6% polyacrylamide gels, stained with ethidium bromide, and visualized under ultraviolet light. A sample was considered positive for *T. vaginalis* if it yielded a 102 base pair band that was cleaved by *Hinfl* to 56 and 46 base pair bands. A typical result is shown in Figure 1.

PCR results were not available to clinicians. All samples were assayed blindly without knowledge of clinical or microbiological findings. Since the PCR analyses were performed on frozen samples, after the women were no longer pregnant or being seen

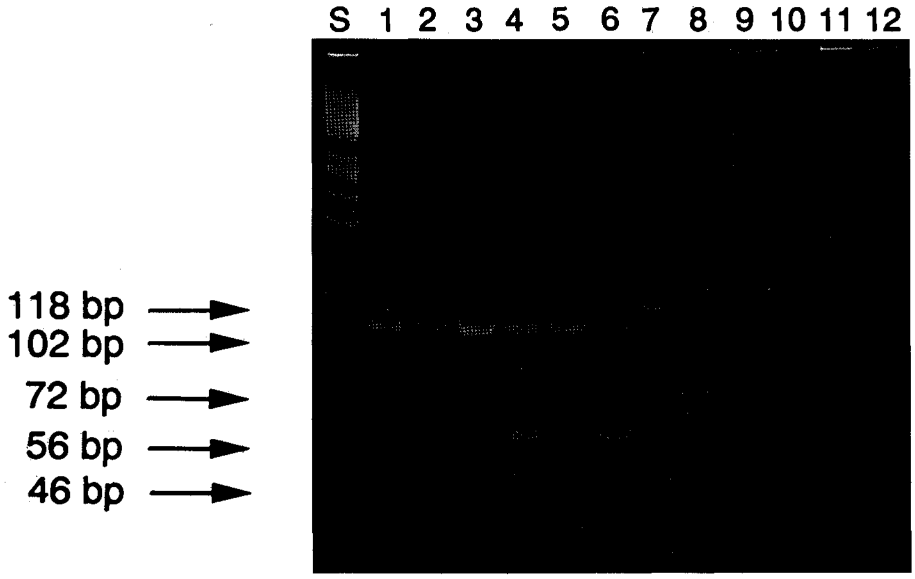

Fig. 1. Detection of *T. vaginalis* in vaginal samples by PCR. Vaginal samples, as well as purified *T. vaginalis* as a positive control, were digested with detergent and proteinase K and heated to lyse the cells. Aliquots were incubated with PCR reaction mixture containing primer pairs specific for the *T. vaginalis* genome. After 40 cycles of sequential incubations, the reaction products were treated with *HinfI* endonuclease, subjected to electrophoresis, and visualized under ultraviolet light after ethidium bromide staining. Samples yielding a 102 base pair product that was cleaved to 56 and 46 base pair products were positive for *T. vaginalis*. **Lane S:** Oligonucleotide base pair standards; odd-numbered lanes: PCR products prior to endonuclease digestion; even-numbered lanes: PCR products after endonuclease digestion. **Lanes 1, 2:** Purified *T. vaginalis*; **lanes 3–6:** 2 vaginal samples positive for *T. vaginalis*; **lanes 7–12:** 3 vaginal samples negative for *T. vaginalis*.

for vaginitis, the subjects were not treated based on PCR findings.

### RESULTS

Among 52 non-pregnant women with symptoms of a vaginal infection, 7 (13.5%) were positive for vaginal *T. vaginalis* by PCR. Six of these 7 women were also positive for *T. vaginalis* by culture in Diamond's medium. The remaining women were all negative for *T. vaginalis*. Bacterial vaginosis was diagnosed in 12 (23.1%) of the women, only 1 of whom was *T. vaginalis* positive by both PCR and culture. Similarly, of the 13 (25.0%) women with a *Candida* infection, only 1 had evidence of *T. vaginalis* by PCR and culture. These results are summarized in Table 1.

None of the 131 pregnant women were positive for *T. vaginalis* by wet mount. However, PCR analysis revealed that 10 (7.6%) of these women harbored *T. vaginalis*.

The times of sample collection, gestational ages at delivery, and pregnancy outcomes of the PCR-positive pregnant women are shown in Table 2.

TABLE 1. Detection of *T. vaginalis* by PCR and culture in women with vaginal infection[a]

| Infection | No. women (%) | No. positive for *T. vaginalis* | |
|---|---|---|---|
| | | PCR | Culture |
| *T. vaginalis* | 7 (13.5%) | 7 | 6 |
| Bacterial vaginosis | 12 (32.1%) | 1 | 1 |
| *Candida* | 13 (25.0%) | 1 | 1 |

[a]Fifty-two women with vaginal symptoms were tested.

Seven of the samples were not obtained until the 3rd trimester; the other 3 samples were obtained during the 13th, 18th, and 25th weeks of gestation. Eight (80%) of the pregnancies were uneventful and delivered at term (>36 weeks), including the woman whose trichomoniasis was detected at the 13th week. Two of the PCR-positive pregnant women delivered preterm at 36 weeks. One of these women delivered a small-for-gestational-age infant.

**TABLE 2. Pregnancy outcome in asymptomatic women positive for *T. vaginalis* by PCR[a]**

| Patient | Gestational age (weeks) | | Outcome |
|---|---|---|---|
| | Sample | Delivery | |
| 1 | 35 | 36 | SGA |
| 2 | 35 | 36 | Normal |
| 3 | 13 | 37 | Normal |
| 4 | 25 | 39 | Normal |
| 5 | 36 | 39 | Normal |
| 6 | 33 | 40 | Normal |
| 7 | 18 | 40 | Normal |
| 8 | 33 | 41 | Normal |
| 9 | 36 | 42 | Normal |
| 10 | 35 | 42 | Normal |

[a]Samples from the posterior fornices of 131 pregnant women were tested for *T. vaginalis* by PCR. SGA = small for gestational age. A normal pregnancy outcome is defined as delivery ≥37 weeks, no intrapartum or postpartum fever, and 5-min Apgar score >7.

### DISCUSSION

Among symptomatic women, PCR was at least as sensitive as culture in modified Diamond's medium in detecting *T. vaginalis*. The PCR assay was consistently negative in women whose symptoms were due to bacterial vaginosis or vaginal candidiasis. The single PCR-positive, culture-negative case most likely represented a *T. vaginalis* isolate that was unable to replicate in culture.[7] Alternatively, the concentration of *T. vaginalis* might have been too low to allow for visualization after a 7-day incubation period in Diamond's medium. PCR analysis can be completed in several hours, as opposed to 7 days for culture. This assay can be readily incorporated into the repertoire of any laboratory that performs PCR for detection of other microorganisms. Timely, definitive diagnosis of *Trichomonas* using PCR would eliminate the need for empiric metronidazole treatment.

Not surprisingly, PCR was superior to wet mount in identifying *T. vaginalis* in asymptomatic pregnant women. Our identification of *T. vaginalis* by PCR in 7.6% of these women indicates that this protozoan may be more prevalent than previously suspected in this population. Since preliminary results suggest that both symptomatic and asymptomatic women with *T. vaginalis* infection suffer increased risks of preterm birth and premature rupture of membranes,[4] methods to increase the detection rate of this organism, and subsequent treatment, may improve pregnancy outcomes in infected women.

PCR analysis may be especially beneficial for those women who are negative for *T. vaginalis* by wet mount but whose symptomatic vaginitis remains unexplained. Further studies are required to confirm the utility of this important diagnostic test as well as to demonstrate the clinical benefits of treating pregnant and non-pregnant women whose trichomoniasis was identified only by PCR.

### ACKNOWLEDGMENTS

These studies were supported in part by a grant from the Eleanor Naylor Dana Charitable Trust. The technical assistant of Yvonne Schaeffer is gratefully acknowledged.

### REFERENCES

1. Hammill HA: *Trichomonas vaginalis*. Obstet Gynecol Clin North Am 16:531–540, 1989.
2. Wolner-Hanssen P, Krieger JN, Stevens CE, Kiviat NB, Koutsky L, Critchlow C, DeRouen T, Hillier S, Holmes KK: Clinical manifestations of vaginal trichomoniasis. JAMA 261:571–576, 1989.
3. Heine P, McGregor JA: *Trichomonas vaginalis*: A reemerging pathogen. Clin Obstet Gynecol 36:137–144, 1993.
4. Cotch MF, Vaginal Infection and Prematurity Study Group: Carriage of *Trichomonas vaginalis* is associated with adverse pregnancy outcome. 30th Intersci Conf Antimicrob Agents Clemother Abst 681, Atlanta, GA, American Society for Microbiology. Oct. 21–24, 1990.
5. Soper DE, Bump RC, Hurt WG: Bacterial vaginosis and trichomoniasis vaginitis are risk factors for cuff cellulitis after abdominal hysterectomy. Am J Obstet Gynecol 163: 1016–1023, 1990.
6. McMillian A: Laboratory diagnostic methods and cryopreservation of trichomonads. In Honigsberg BM (ed): Trichomonads Parasitic in Humans. New York: Springer-Verlag, pp 297–310, 1991.
7. Draper D, Parker R, Patterson E, Jones W, Beutz M, French J, Borchardt K, McGregor J: Detection of *Trichomonas vaginalis* in pregnant women with the InPouch TV culture system. J Clin Microbiol 31:1061–1068, 1993.
8. Riley DE, Roberts MC, Takayama T, Kriegor JN: Development of a polymerase chain reaction-based diagnosis of *Trichomonas vaginalis*. J Clin Microbiol 30:465–472, 1992.

## References

[PDF_00291] Hammill H. A. (1989). Trichomonas vaginalis.. Obstet Gynecol Clin North Am.

[PDF_00297] Heine P., McGregor J. A. (1993). Trichomonas vaginalis: a reemerging pathogen.. Clin Obstet Gynecol.

[PDF_00316] Riley D. E., Roberts M. C., Takayama T., Krieger J. N. (1992). Development of a polymerase chain reaction-based diagnosis of Trichomonas vaginalis.. J Clin Microbiol.

[PDF_00304] Soper D. E., Bump R. C., Hurt W. G. (1990). Bacterial vaginosis and trichomoniasis vaginitis are risk factors for cuff cellulitis after abdominal hysterectomy.. Am J Obstet Gynecol.

[PDF_00293] Wølner-Hanssen P., Krieger J. N., Stevens C. E., Kiviat N. B., Koutsky L., Critchlow C., DeRouen T., Hillier S., Holmes K. K. (1989). Clinical manifestations of vaginal trichomoniasis.. JAMA.

